# Improved Hole Injection by Enhancing Electron Extraction of Solution-Processed MoO_*x*_ in Quantum Dot Light-Emitting Diodes

**DOI:** 10.1021/acsami.5c01875

**Published:** 2025-06-10

**Authors:** Jing Jiang, Ting Ding, Hui Bao, Yin-Man Song, Meng-Wei Wang, Hang Liu, Zhi-Sheng Wu, Zhen-Dong Lian, Hai-Zheng Zhong, Hong-Chao Liu, Shu-Ming Ren, Yang Li, Pei-Li Gao, Kar Wei Ng, Shuang-Peng Wang

**Affiliations:** † Institute of Applied Physics and Materials Engineering, 59193 University of Macau, Taipa, Macao SAR 999078, China; ‡ MIIT Key Laboratory for Low-Dimensional Quantum Structure and Devices, School of Materials Science & Engineering, 47833 Beijing Institute of Technology, Beijing 100081, China; § Fujian Science & Technology Innovation Laboratory for Optoelectronic Information of China, Fuzhou 350108, China; ∥ Poly Optoelectronics Tech. Ltd, Jiangmen 529020, China

**Keywords:** hole injection layer, MoO_*x*_, electron extraction, UV-ozone, QLED

## Abstract

Hole injection layers (HILs) are pivotal for the performance of quantum dot light-emitting diodes (QLEDs), with solution-processed inorganic HIL materials being a primary means for the commercial application of QLEDs. Transition metal oxides (TMOs), due to their excellent stability, have been widely employed as inorganic HILs by thermal evaporation in QLEDs; however, the hole injection ability of solution-processed TMO film necessitates further enhancement owing to inferior film quality. In this study, a solution-processed molybdenum oxide (MoO_*x*_) film was used as a HIL, and its hole injection ability in the QLED was improved by tuning the oxygen states. Oxidation treatments on the MoO_*x*_ layer can effectively mitigate oxygen vacancies, and consequently, the conduction band minimum (CBM) of MoO_*x*_ is elevated, which enhances the hole injection through easier electron extraction from the highest occupied molecular orbitals (HOMO) of the hole transport layer. The MoO_*x*_-based red QLED exhibits significantly longer working lifetimes (T50@100 cd·m^–2^ of ∼66,892 h) and comparable current efficiencies (13.7 cd·A^–1^) compared to the Poly­(3,4-ethylenedioxythiophene)-poly­(styrenesulfonate) (PEDOT:PSS)-based QLEDs. Our research not only proposes a promising approach to high-performance MoO_*x*_-based QLEDs but also paves the way for further applications of TMOs in QLEDs.

## Graphical abstract

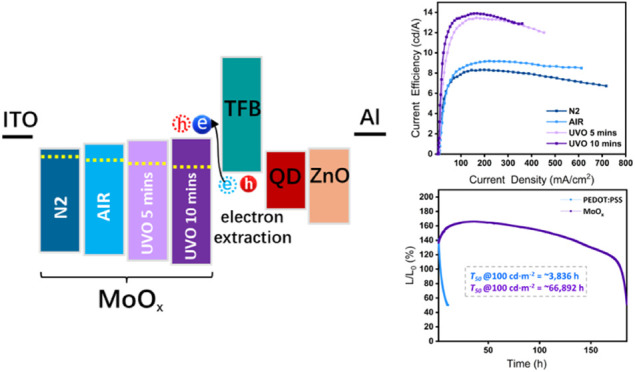

## 1 Introduction

Quantum Dot Light-Emitting Diodes (QLEDs) promise a new generation of display industry due to their distinctive characteristics, including highly color-pure emissions, tunable emission wavelengths, and a wide gamut.^1–5^ More importantly, the solution-processable QLEDs promise a low-cost solution for large-scale display, which facilitates further commercialization of QLEDs.^6,7^ Although QLEDs featuring a hybrid organic–inorganic structure have exhibited high efficiency, the batch instability and vulnerability to the environment of organic materials still impede the application of QLEDs.^8,9^ For example, Poly­(3,4-ethylenedioxythiophene)-poly­(styrenesulfonate) (PEDOT:PSS), a widely utilized hole injection layer (HIL) in QLEDs, has long been presumed to adversely affect device lifetime owing to its acidic and hygroscopic properties.^10–12^ Among the intensely studied alternatives of PEDOT:PSS, transition metal oxides (TMOs), including vanadium oxides (V_2_O_5_),^13^ molybdenum oxides (MoO_*x*_)^14,15^ and tungsten oxides (WO_*x*_),^16^ show great promise as their intrinsic stability and tunability of electronic properties.

Due to the low cost, high capacity, and controllable chemical compositions, MoO_*x*_ is the most promising candidate among the mentioned TMOs.^17,18^ The MoO_*x*_ thin films have been proven to be strongly n-type semiconductors with a high electron affinity (6.70 eV) and ionization energy (9.68 eV). Consequently, the hole injection process occurs through electron extraction from the highest occupied molecular orbitals (HOMO) of the organic hole transport layer (HTL) into the conduction band of MoO_*x*_.^19^ Furthermore, the oxygen stoichiometry of MoO_*x*_ modulates both the bandgap and work function (WF) of MoO_*x*_,^20^ with an upward shift in the conduction band minimum (CBM) resulting in a reduced energy barrier between the CBM of MoO_*x*_ and the HOMO of organic HTL. Extensive computational studies also reveal that MoO_*x*_ exhibits exceptionally high WF and low-lying CBM. These unique electronic properties establish favorable energy-level alignment with the HOMO of conventional organic HTL materials, thereby highlighting the significant potential of MoO*_x_* for efficient hole injection applications.^21–23^

Thermal evaporation has successfully achieved high-quality, high-crystallinity, continuous, uniform, and thickness-controlled MoO_*x*_, demonstrating its feasibility in inverted QLEDs compared to other TMOs.^24–26^ To align with the low-cost inkjet printed QLED technology and facilitate further commercialization, it is necessary to develop a solution-processed MoO_*x*_ hole injection layer (HIL) with excellent carrier injection ability. Vu and co-workers reported a solution-processed MoO_*x*_-based HIL in green QLEDs.^27^ In this work, they prepared the MoO_*x*_ film by the decomposition of a solution of ammonium molybdate tetrahydrate at 80 °C under ambient conditions, and the stable QLED achieved a maximum current efficiency (CE) of 10.8 cd·A^–1^ at a low turn-on voltage of 2.2 V. Zheng et al. fabricated all solution-processed flexible QLEDs with modified MoO_*x*_ films showing a superior performance of 5.46 cd·A^–1^ to the conventional PEDOT:PSS-based QLEDs.^28^ However, due to the mixed Mo valence states, the solution-processed MoO_*x*_ films usually demonstrate inferior hole injection capabilities,^27,29^ which is incompatible with the HTLs.^30,31^ It is well-known that increasing oxygen contents in MoO_*x*_ can increase the valence states of Mo atoms. This leads to decreased electrons in the Mo *d* orbitals, which alters the electronic conductivity and raises the CBM of MoO_*x*_ films.^20,23^ The oxidation method has been successfully implemented in MoO_*x*_-based solar cells and two-dimensional materials.^32,33^ However, the influence of the oxidation state changes of spin-coated MoO_*x*_ films on all solution-processed QLEDs has not been thoroughly researched.

In this work, we demonstrate a strategy to enhance the efficiency and operational stability of all solution-processed MoO_*x*_-based QLEDs by optimizing the hole injection properties of a MoO_*x*_ HIL through the control of Mo valence states. By subjecting the MoO_*x*_ films to various oxidation environments—such as nitrogen, air, and active oxygen species, we systematically modulate the oxidation states of Mo, which directly influence the energy band alignment and charge injection dynamics. This optimization facilitates efficient hole injection by promoting electron extraction from the HOMO of the HTL. Furthermore, we investigate the effects of UV light and ozone (O_3_) on the chemical and electronic properties of MoO_*x*_ films, revealing that O_3_ plays a dominant role in tuning the oxidation states and improving the hole injection efficiency. The resulting all solution-processed red QLEDs with UV-ozone-treated MoO_*x*_ HIL achieve a maximum current efficiency (CE) of 13.7 cd·A^–1^, comparable to that of conventional PEDOT:PSS-based devices. Remarkably, these devices exhibit exceptional operational stability, with an average lifetime *T*_50_ (the time required for the initial luminance (*L*_0_) to decrease to 50%) of approximately 66,892 h at 100 cd·m^–2^, significantly outperforming PEDOT:PSS-based QLEDs (T50@100 cd·m^–2^ of ∼3836 h). This study not only highlights the potential of MoO_*x*_ as a high-performance HIL material but also provides a scalable and cost-effective approach for the development of solution-processed optoelectronic devices.

## 2 Experimental Section

### 2.1 Materials

Poly [(9,9-dioctylfluorene-*co*-*N*-(4-(3-methylpropyl)) diphenylamine)] (TFB) was purchased from Sigma-Aldrich. MoO_*x*_ nanoparticles, CdSe/ZnS QDs, and ZnO nanoparticles were purchased from Guangdong Poly Optoelectronics Co. Ltd., China. Except for the CdSe/ZnS QDs, which were diluted, other chemicals were used as received without any further purification.

### 2.2 Device Fabrication

QLEDs were fabricated on patterned indium tin oxide (ITO) glass substrates (Wuhu Jinghui Electronic Technology Co. Ltd.), which were cleaned successively using deionized water, toluene, acetone, ethanol, and isopropanol under an ultrasonic bath for 15 min. Then, the ITO glass substrates were dried with nitrogen and exposed to UV-ozone for 30 min. The device structure of solution-processed red QLEDs is ITO/MoO_*x*_/TFB/QDs/ZnO/Al. The MoO_*x*_ (15 mg·mL^–1^ in *n*-butanol) layer was spin-coated on ITO substrate at 3000 rpm for 40 s and then annealed the film at 150 °C for 10 min (In this work, we mainly have 4 samples, and one of them MoO_*x*_ layer was spin-coated and annealed both in nitrogen atmosphere, while the MoO_*x*_ layer of other three were all spin-coated and annealed in the air). Then, two of the MoO_*x*_ films, which were spin-coated and annealed in the air, were treated with UV-ozone (BZS250GF-TC) for 5 and 10 min, respectively. Next, all the samples were transferred to an N_2_-filled glovebox for the deposition of the following layers. The TFB (8 mg·mL^–1^ in chlorobenzene) layer was spin-coated onto MoO_*x*_ at 3000 rpm for 40 s, followed by annealing at 120 °C for 10 min. QDs (20 mg·mL^–1^ in *n*-octane) were spin-coated onto TFB at 3000 rpm for 40 s. The ZnO layer was then deposited from its ethanol solution at 3000 rpm for 40 s and annealed at 60 °C for 40 s. Subsequently, all samples were sent into a high vacuum deposition system, and then, an evaporated, 90 nm thick aluminum was performed as the cathode was deposited at a rate of 0.25 nm·s^–1^.

### 2.3 Characterization

The morphology of MoO_*x*_ films was captured with a scanning electron microscope (SEM, Sigma FE-SEM, Zeiss Corporation, Germany). The atomic force microscopy (AFM) images of MoO_*x*_ films were captured with the device of Dimension Icon, Bruker, Germany. The UV-Ozone treatments were conducted by a UV-ozone cleaner BZS250GF-TC (185 and 254 nm, 250 W). The UV–vis absorption spectrum was obtained by a UV–vis/NIR spectrophotometer (UV-2600, Shimadzu, Japan). The Raman spectra were researched with a Confocal Laser Raman Spectrometer (LabRAM HR Evolution, HORIBA, Japan). X-ray photoelectron spectroscopy (XPS) spectra were measured by ESCA Lab220I-XL. Ultraviolet photoelectron spectroscopy (UPS) spectra were performed using an ESCA Lab220I-XL. The source meter (Keithley 2400) and the luminance meter (Minolta LS-110) were employed to measure the current density–voltage–luminance characteristics.

## 3 Results and Discussion

The MoO_*x*_ nanoparticle solution exhibits a Prussian blue color (Figure 1a­(i)), originating from the metastable state of mixed Mo^5+^ and Mo^6+^ cation oxidation states.^20,34^ The solution-processed MoO_*x*_ film is fabricated onto a patterned indium tin oxide (ITO) glass by spin-coating in the air, which appears light blue (Figure 1a­(ii)) and shows a uniform and continuous morphology (Figure 1a­(iii)) with a root-mean-square roughness of 1.56 nm (Figure S1). Then the MoO_*x*_ films are annealed in N_2_ (no oxygen), air (with oxygen), and UV-ozone (with active oxygen), respectively (Figure 1b), which are labeled N2, AIR, UVO-5, and UVO-10. The color transitions from dark blue (N2) to transparent (UVO-10) as the MoO_*x*_ films undergo increased oxidation. This phenomenon is confirmed by ultraviolet–visible absorption spectroscopy (Figure S2a). Specifically, the N2 sample exhibited significant absorption in the 450–600 nm range, which aligns with the absorption characteristics of Mo^5+^ cations.^35^ In contrast, the absorption of the AIR sample was notably diminished. As the degree of oxidation increased, the absorption became invisible in the UVO-5 and UVO-10 samples. The bandgap also becomes greater upon the increased content of Mo^6+^ cations (Figure S2b). The increase stems from the tighter binding of delocalized electrons to the Mo^6+^ core cation, which effectively diminishes the gap states between the conduction and valence bands of MoO_*x*_.^20,32^ The X-ray diffraction spectra (Figure S3) also confirmed the XPS results. The results above collaboratively confirm that the Mo/O ratio and the valence state of Mo in the MoO_*x*_ film can be modulated by annealing the film in environments with different contents of oxygen, thereby effectively regulating the film properties.

**Figure 1:**
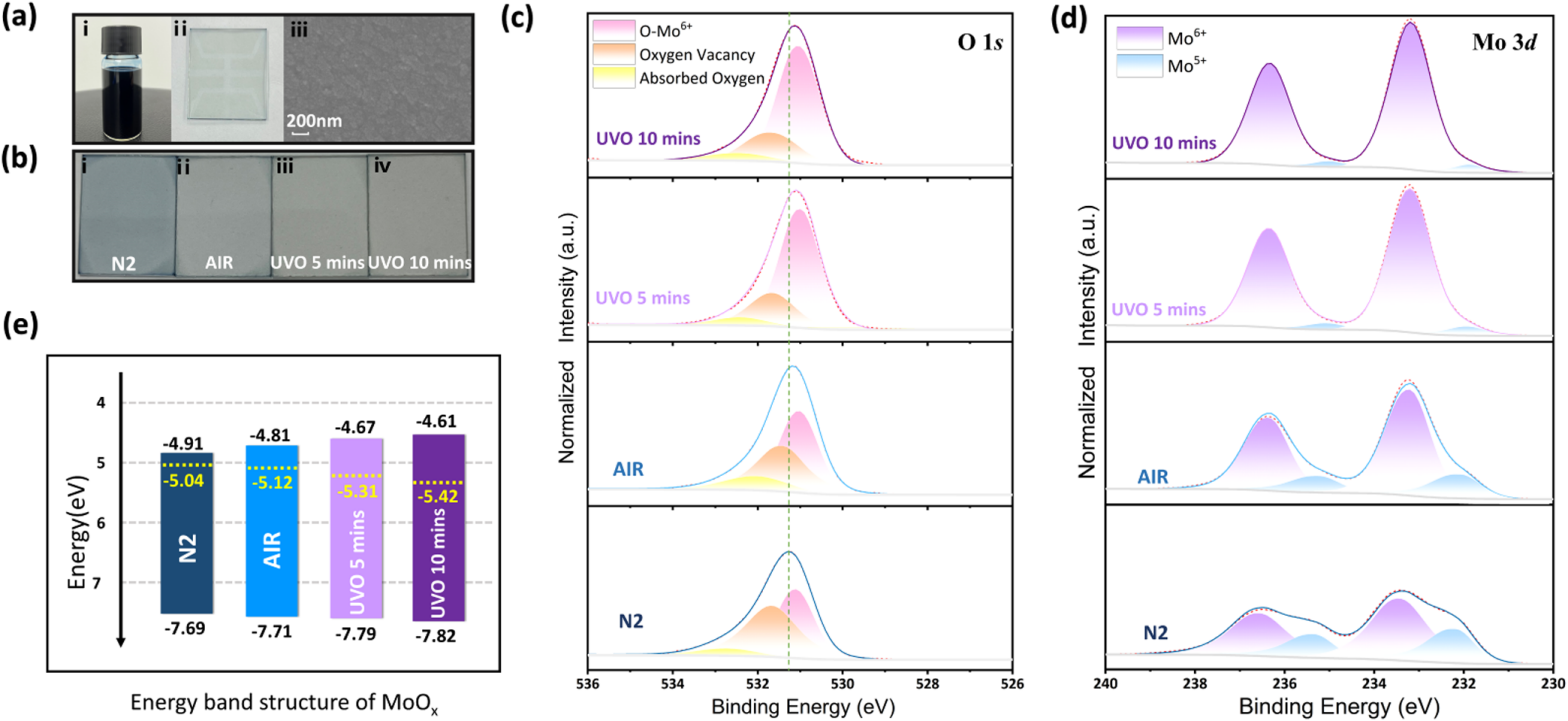
Tune the energy band structure of MoO_*x*_ films. (a) (i) MoO_*x*_ *n*-butanol solution, (ii) spin-coating MoO_*x*_ solution on ITO glass, (iii) Surface morphology of MoO_*x*_ films by SEM, (b) MoO_*x*_ films treated under different conditions (i) annealed in N_2_, (ii) annealed in the air, (iii) annealed in the air and treated with UV-ozone for 5 min, (iv) annealed in the air and treated with UV-ozone for 10 min, (c) XPS of O 1*s* core level of MoO_*x*_ films, (d) XPS of Mo 3*d* core level of MoO_*x*_ films, and (e) energy-level diagram of MoO_*x*_ films, the yellow dash line labels the Fermi level.

X-ray photoelectron spectroscopy (XPS) measurements were conducted to unravel the specific oxygen stoichiometry of the MoO_*x*_ films with different oxygen treatments. The deconvolution of the O 1s peaks for MoO_*x*_ films (Figure 1c) results in three contributions: lattice oxygen (O_L_) at 531.1 eV, oxygen vacancies (V_O_) at 531.7 eV, and chemisorbed oxygen species (O_C_) at 532.8 eV.^36–42^ Table S1 gives the quantitative content ratios of different oxygen species. It can be observed that as oxidation increases, the O_L_ content increases while the V_O_ concentration decreases. UV-Ozone treatment, due to its strong oxidative capability, significantly enhances the level of O_L_ and reduces the V_O_ content. However, it readily reaches oxidation saturation, resulting in only minor changes in the O_L_ (V_O_) between UVO-5 min and UVO-10 min samples. Figure S4 more clearly illustrates this trend, which indicates that oxidation can significantly reduce the presence of V_O_ in MoO_*x*_, causing the O 1*s* peak to become narrower and shift to lower energy. Meanwhile, the changes in Mo 3*d* doublets for Mo^5+^ and Mo^6+^ show similar trends. As shown in Figure 1d, all MoO_*x*_ films have peaks located at 236.2 and 233.1 eV signifying Mo^6+^ states,^14,43^ and in the N2-MoO_*x*_ film, there are noticeable shoulders at 235.4 and 232.2 eV, which correspond to the Mo^5+^ states. With the increasing oxidation, the Mo^5+^ signal gradually weakens until it disappears, showing that Mo^5+^ is transformed into Mo^6+^, which is consistent with the changes in oxygen. XPS results confirm that oxidation in an oxygen-rich environment can effectively inhibit the formation of V_O_ and align the valence state of Mo in the MoO_*x*_ films.

The change in the oxidation states of MoO_*x*_ leads to different work functions and band structures. Figure 1e shows the energy band structures of MoO_*x*_ films determined by the ultraviolet photoelectron spectroscopy results (Figure S5) and absorption spectra (Figure S2). It can be observed that the electron affinity decreases obviously with the increase of oxidation states of MoO_*x*_, while the work function slightly increases. The elevated CBM can alter the band alignment in the QLED, thereby affecting the hole injection ability. Additionally, the difference between the CBM and Fermi levels increased from approximately 0.13–0.81 eV, indicating that the N2-MoO_*x*_ possesses a higher electron concentration in the conduction band, resulting in lower series resistance in QLEDs. This higher electron filling level can be attributed to gap states induced by the V_O_ and mixed Mo valence states, which gradually diminish during the oxidation processes. These findings further corroborate the XPS results.

To verify the MoO_*x*_-HIL performance, red emission QLEDs ITO/MoO_*x*_/Poly­[(9,9-dioctylfluorenyl-2,7-diyl)-*co*-(4,4′-(*N*-(4-s-butylphenyl)­diphenylamine)] (TFB)/QDs/ZnO/Al) are constructed (Figure 2a). The solution-processed MoO_*x*_ HILs are fabricated under identical conditions and possess similar thicknesses. The QLEDs emit a bright red light centered at 625 nm under forward bias, which aligns with the photoluminescence characteristics of the QD (Figure S6). As presented in Figure 2b, the upward-shifted CBM induced by the oxidation treatment lowers the electron extraction barrier, thereby enhancing hole injection efficiency into TFB in operational devices.^19,44,45^ This enhancement is anticipated to result in a proportional increase in both current and luminance after the device turns on. However, the increased distance between the CBM and the Fermi level lowers the electron concentration in the conduction band of MoO_*x*_. This increases the series resistance in QLEDs, resulting in a reduced effective voltage across the active region, which consequently increases the turn-on voltage and decreases the current passing through the device. As depicted in Figure 2c, the N2-MoO_*x*_ QLED exhibits a higher current and a lower turn-on voltage, attributed to the lower series resistance. In the case of partially oxidized MoO_*x*_ (AIR-MoO_*x*_ QLED), the initial current is comparable to that of N2-MoO_*x*_ QLED; however, after turn-on, the current drops significantly, and the turn-on voltage exceeds that of the N2-MoO_*x*_ QLED. With further oxidation, the currents of UVO-5 and UVO-10 QLEDs decrease even more markedly. Notably, the luminance does not decline proportionally with the current reduction, suggesting improved hole injection efficiency. Consequently, the UVO-10 QLED achieves a high CE of 13.7 cd·A^–1^, which represents a substantial enhancement of 67 and 51% compared to the N2-QLED (8.2 cd·A^–1^) and AIR-QLED (9.1 cd·A^–1^), respectively (Figure 2d). These experimental results align well with our initial hypothesis. However, excessively prolonged UV-ozone treatment can degrade the performance of QLEDs (Figure S7). This is likely due to overoxidation and potential structural damage to the MoO_*x*_ layer.

**Figure 2:**
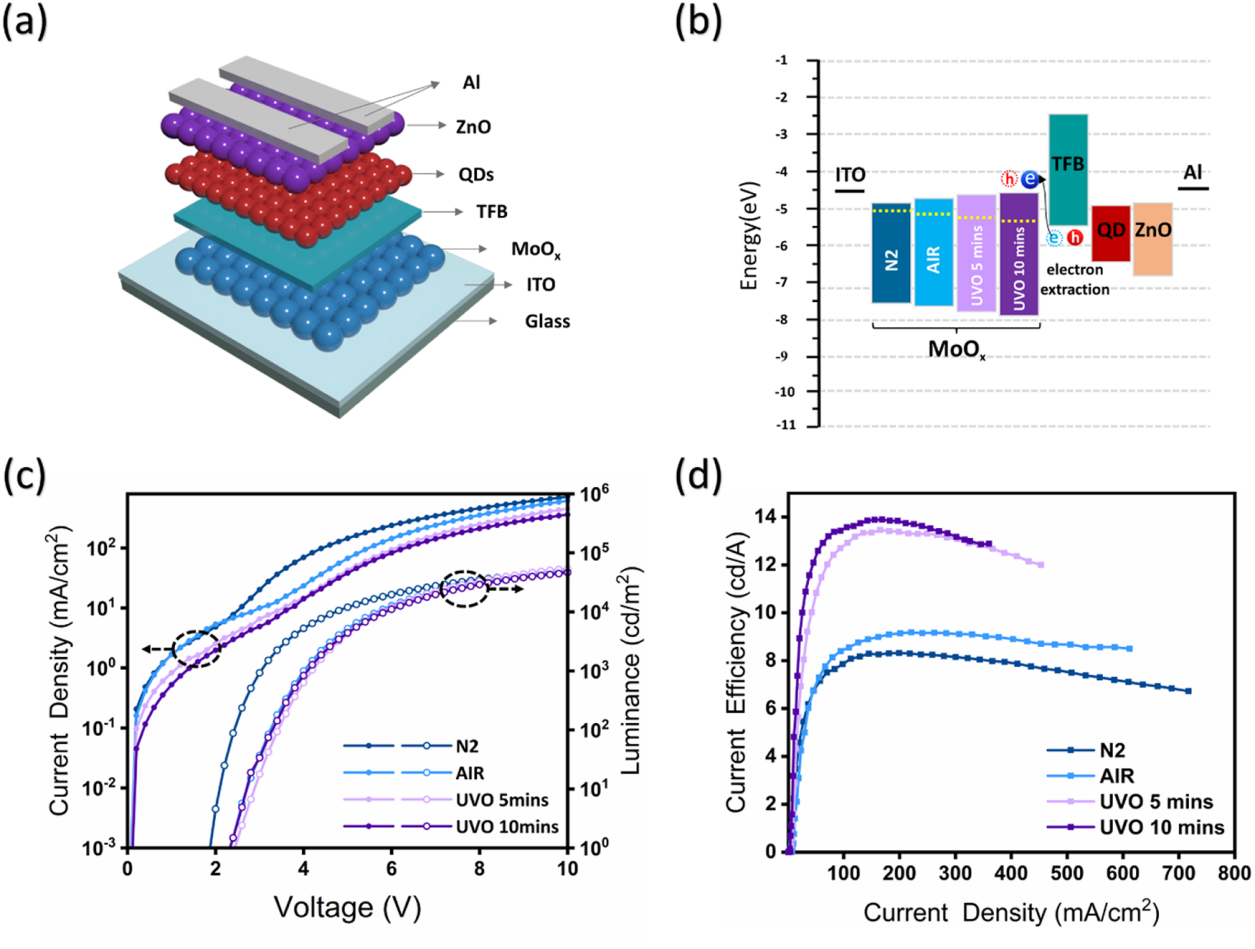
Improved hole injection efficiency with the UV-ozone treatment on the solution-processed MoO_*x*_-HIL. (a) Schematic of the device structure, (b) Energy band diagram of red QLEDs with different MoO_*x*_ HILs, (c) Current density (J)-voltage (V)-luminance (L), and (d) current efficiency (CE)-J characteristics of red QLEDs.

To further verify whether the alteration in the oxidation states of MoO_*x*_ is predominantly induced by UV light and O_3_, or a combination of both, the MoO_*x*_ films were subjected to separate treatments involving UV irradiation and O_3_ exposure. Specifically, UV treatment was conducted using a 365 nm UV lamp within a nitrogen-filled glovebox, while O_3_ was generated using an ozone generator for the respective treatment. In Figure 3a, the current density profile of the QLED with an O_3_-treated MoO_*x*_ HIL exhibits similarities to that of the UVO-10 QLED. However, the O_3_-treated QLED exhibits a lower current density compared to the UVO-10 QLED, which can be attributed to insufficient oxidation in the O_3_-only treated MoO_*x*_. Although the O_3_ treatment eliminates the influence of bandgap states in MoO_*x*_, leading to an increase in series resistance, the hole injection capability has not yet reached the level of the UVO-10 QLED. The slightly lower turn-on voltage of the O3 QLED compared to the UVO-10 QLED suggests that the gap states are not completely eliminated. Furthermore, the trends in current density and luminance after turn-on, as reflected by CE (Figure 3b), indicate that the efficiency of the O_3_-treated sample is slightly lower than that of the UVO-10 QLED. This confirms that the hole injection capability of the O_3_-treated HIL is not as effective as that of the UVO-10-treated HIL. As demonstrated in Figure S8, prolonging the O_3_ treatment time brings the J-V-L characteristics of the O3 QLED closer to those of the UVO-10 QLED. However, key parameters, such as the turn-on voltage and CE, show no significant differences, indicating that the extended oxidation treatment does not substantially enhance the hole injection capability and overall device performance.

**Figure 3:**
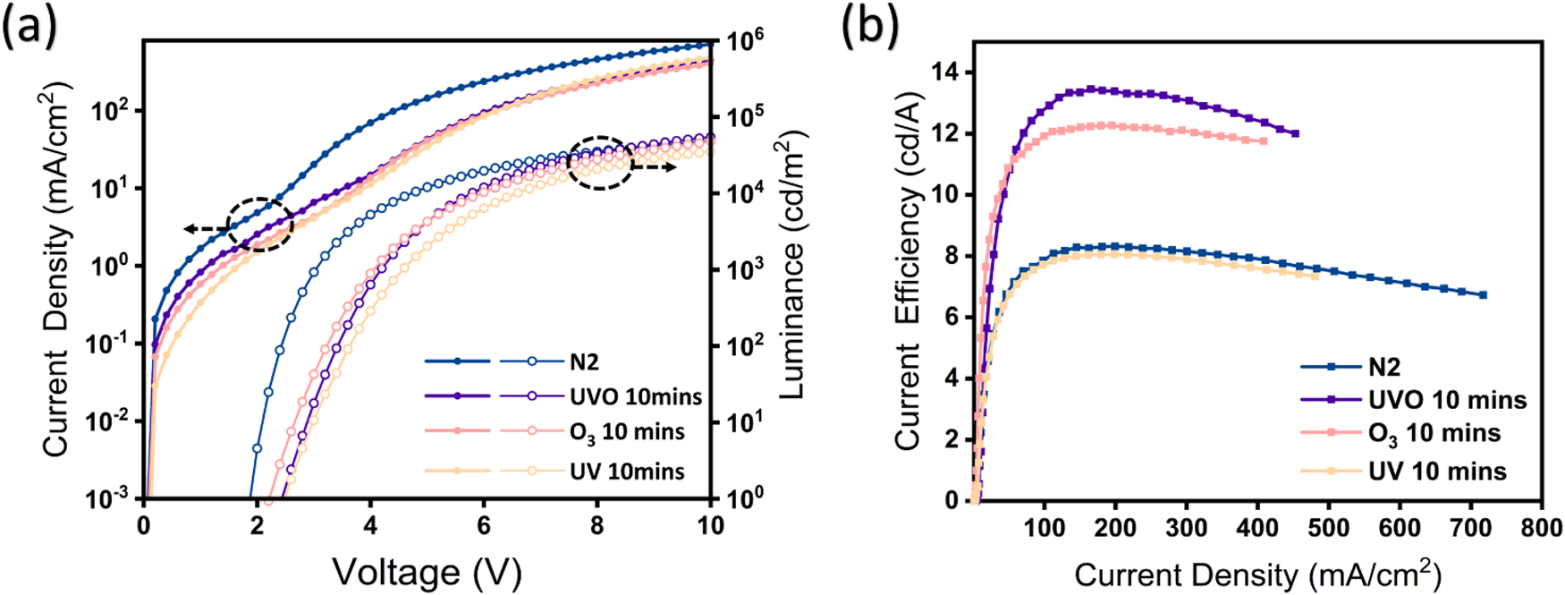
Performances of QLEDs with solution-processed MoO_*x*_-HIL with O_3_ or UV light (365 nm) treatments, (a) J-V-L, (b) CE-J characteristics.

On the other hand, the QLED with UV-treated MoO_*x*_ exhibits a faster current rise trend in the turn-on region compared to others, but its luminance is significantly lower (Figure 3a). This trend is more intuitively reflected in the CE (Figure 3b). Furthermore, extending the UV treatment time significantly reduces both the current density and the luminance of the device, leading to a further decline in CE (Figure S9). The CE statistics of MoO_*x*_-based QLEDs are shown in Figure S10. These observations indicate that while the high-energy photons from UV treatment can influence the properties of MoO_*x*_ HILs, they do not enhance the hole injection efficiency. Therefore, oxidation is the key to enhancing the performance of MoO_*x*_ HILs. However, the surface-to-interior oxidation mechanism of the O_3_ treatment alone is insufficient to fully drive the reaction. In contrast, UV treatment provides the necessary energy to break internal bonds, facilitating the oxidation process. Nevertheless, prolonged UV exposure can damage the internal structure of MoO_*x*_, leading to degradation in HIL performance.

We also compare the performance of MoO_*x*_-based QLEDs with that of conventional devices utilizing PEDOT:PSS as the HIL. As observed in Figure 4a, the turn-on voltage of MoO_*x*_-based QLEDs is 2.5 V, slightly higher than the 2 V observed for PEDOT:PSS-based devices. This difference can be attributed to the higher resistance of MoO_*x*_ compared to PEDOT:PSS. Nonetheless, the maximum CE of MoO_*x*_-based QLEDs reaches 13.7 cd·A^–1^, which is comparable to that of PEDOT:PSS devices (Figure 4b). Notably, the MoO_*x*_-based devices demonstrate significantly improved operational stability, with an average *T*_50_ lifetime of 189.2 h at an initial luminance of 5000 cd·m^–2^, far exceeding the 10.9 h observed for the PEDOT:PSS-based devices (Figure 4c). The lifetime of a QLED device can be estimated using the following formula

$$L_{0}^{n}\times T_{50}=\mathrm{constant}$$

where *L*_0_ is the initial luminance, and *n* is the acceleration factor. Based on the literature, *n* typically ranges from 1.5 to 2.^46^ Here, a low value of *n* = 1.5 is applied. Using the formula, the QLEDs with MoO_*x*_ HILs demonstrate an average *T*_50_ lifetime of 66,892 h at 100 cd·m^–2^. This exceptional stability can be attributed to the following factors: (a) The inherent stability of MoO_*x*_. Unlike PEDOT:PSS, which is acidic and can corrode the ITO anode, MoO_*x*_ is chemically inert and exhibits excellent thermal and environmental stability, thereby preventing performance degradation under the operation process. (b) The enhanced hole injection ability. The optimized oxidized MoO_*x*_ HIL enables efficient hole injection, leading to a balanced charge carrier and reduced Joule heating. These advantages highlight the great potential of solution-processed MoO_*x*_ as a viable alternative to PEDOT:PSS in QLED applications, particularly for achieving long operational lifetimes and high performance.

**Figure 4:**
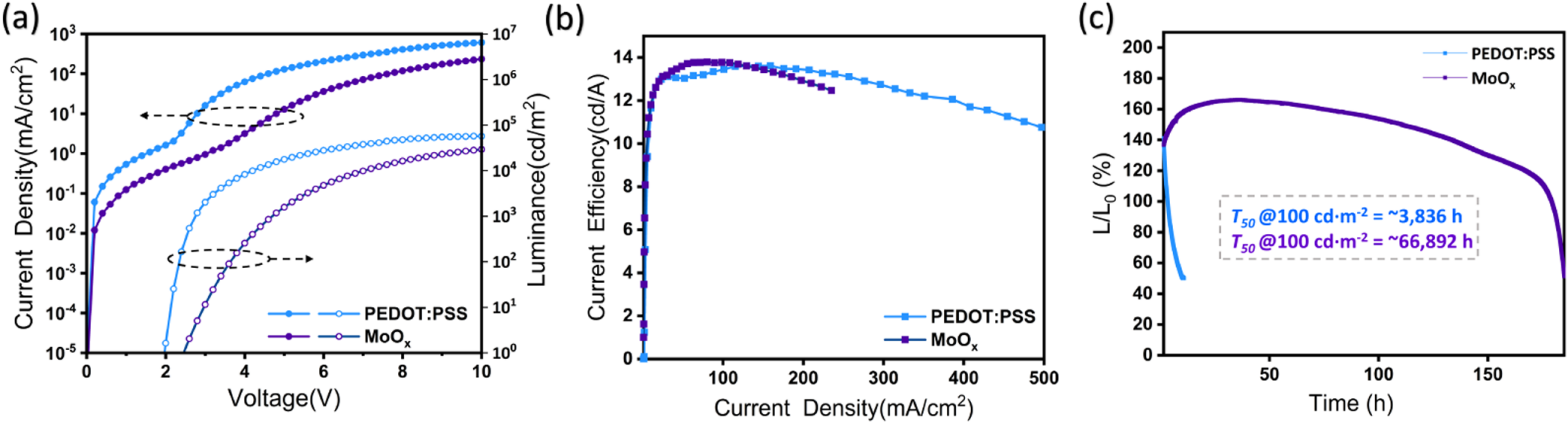
Device characteristics of all solution-processed QLEDs with MoO_*x*_-HIL treated by UV-ozone for 10 min. (a) J-V-L of devices with MoO_*x*_ or PEDOT:PSS as HIL, (b) CE-J, (c) Operational stability data (initial luminance: 5000 cd·m^–2^).

## 4 Conclusions

In conclusion, we have demonstrated that the hole injection efficiency of solution-processed MoO_*x*_-based QLEDs can be significantly improved by increasing the oxidation states of the MoO_*x*_ films. Through a combination of air and UV-ozone treatments, we effectively tune the Mo ions to higher oxidation states, which elevates the CBM of MoO_*x*_ and enhances its hole injection capability. This optimization results in a high CE of 13.7 cd·A^–1^ for MoO_*x*_-based QLEDs, rivaling the performance of PEDOT:PSS-based devices. Additionally, the MoO_*x*_-based QLEDs exhibit outstanding operational stability, with an average *T_50_* lifetime of approximately 66,892 h at 100 cd·m^–2^, far exceeding the ∼3836 h observed for PEDOT:PSS-based devices. Our findings underscore the critical role of O_3_ in the UV-ozone treatment process, as it primarily drives oxidation state modulation and performance enhancement. This work not only presents a simple and cost-effective method for tuning the chemical properties of solution-processed MoO_*x*_ but also opens new avenues for the application of TMOs in printed optoelectronic devices.

## Supplementary Material



## Notes

Characteristics of MoO_*x*_ (AFM images, absorption spectra, XPS, UPS), PL of QDs, and device characteristics of QLEDs with different MoO_*x*_ HILs (PDF)
