# Supplementary material for: Improved Hole Injection by Enhancing Electron Extraction of Solution-Processed MoO x in Quantum Dot Light-Emitting Diodes
Source: ACS Appl Mater Interfaces. 2025 Jun 10;17(25):37185–92. doi: 10.1021/acsami.5c01875 (PMC12203470; doi:10.1021/acsami.5c01875)
Supplement: Supplementary file 1 [file am5c01875_si_001.pdf]

Supporting Information

Improved Hole Injection by Enhancing Electron Extraction of Solution-Processed MoO_x in Quantum Dot Light-emitting Diodes

Jing Jiang[1], Ting Ding[1], Hui Bao[2], Yin-Man Song[1], Meng-Wei Wang[1], Hang Liu[1], Zhi-Sheng Wu[1], Zhen-Dong Lian[1], Hai-Zheng Zhong[2], Hong-Chao Liu[1], Shu-Ming Ren[4], Yang Li[3, 4*], Pei-Li Gao[1*], Kar Wei Ng[1*], Shuang-Peng Wang[1*]

1 Institute of Applied Physics and Materials Engineering, University of Macau, Taipa, Macao SAR 999078, China

2 MIIT Key Laboratory for Low-Dimensional Quantum Structure and Devices, School of Materials Science & Engineering, Beijing Institute of Technology, Beijing 100081, China

3 Fujian Science & Technology Innovation Laboratory for Optoelectronic Information of China, Fuzhou, 350108, China

4 Poly Optoelectronics Tech. Ltd, Jiangmen 529020, China

* Corresponding author:

Pei-Li Gao (peiligao@um.edu.mo),

Yang Li (liyang@fjoel.cn),

Kar Wei Ng (billyng@um.edu.mo),

Shuang-Peng Wang (spwang@um.edu.mo, +853 88224048).

Material and device characteristics:

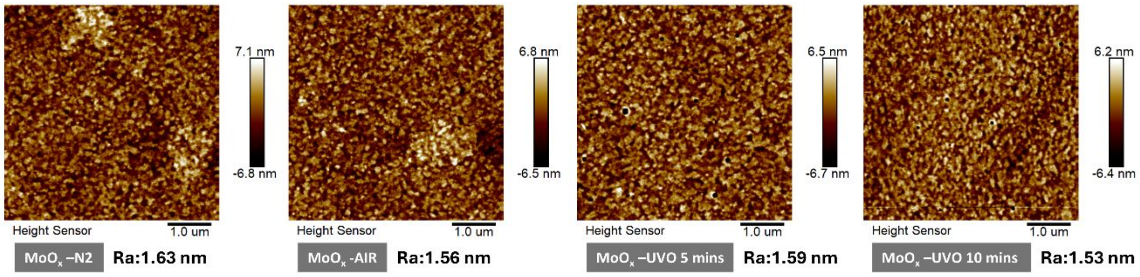

Figure S1. AFM measurements of MoO_x layers with different treatments.

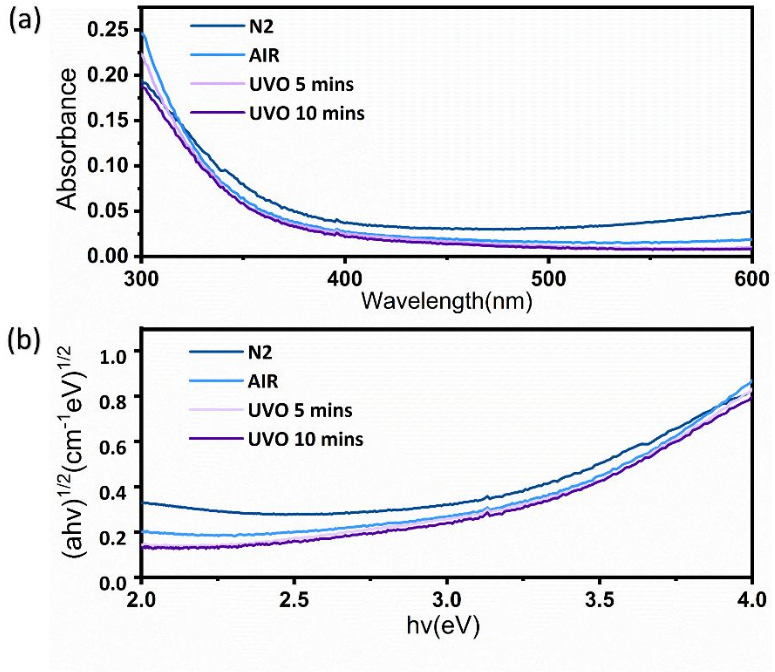

Figure S2. (a) the absorption spectra of MoO_x films, (b) the corresponding Tauc plot of $(\alpha h\nu)^{1/2}$ of the films in Figure 1b. The bandgap of MoO_x films increases with the increase of oxygen states.

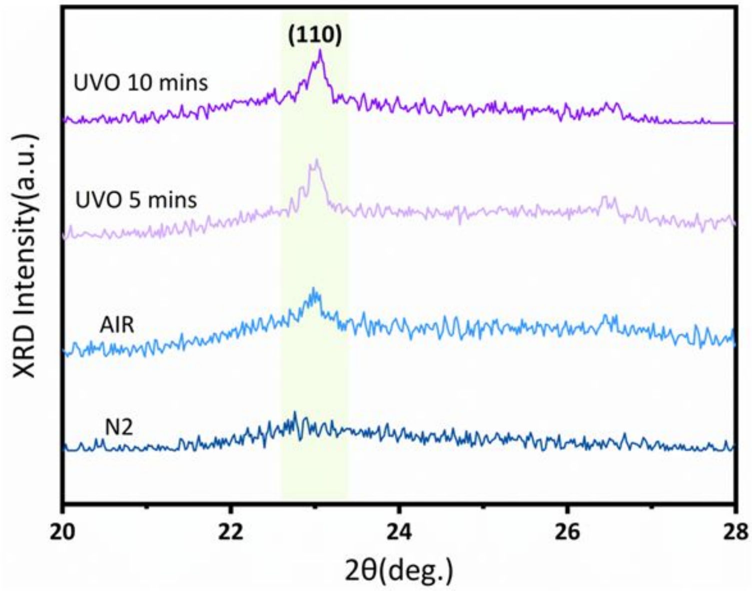

Figure S3. XRD spectra of MoO_x films in Figure 1b. The (110) peaks narrow as the MoO_x films become more oxidized, indicating enhanced formation of MoO_3 as oxidation proceeds.

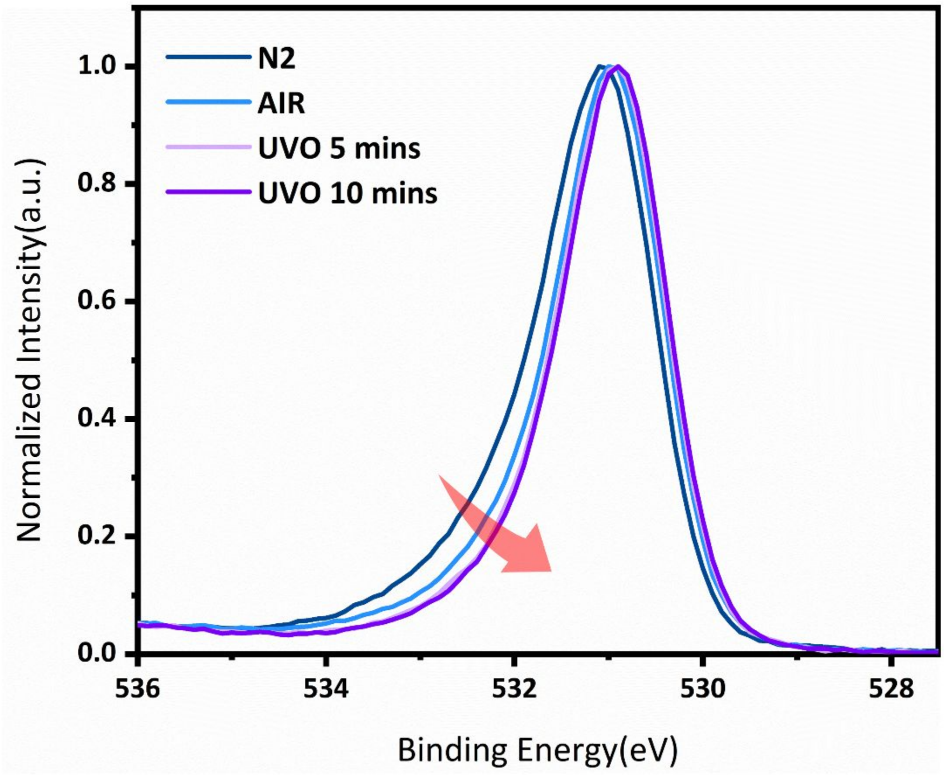

Figure S4. O 1*s* core level of MoO_x films in Figure 1b. The peaks of oxygen vacancies and absorbed oxygen species weaken as the film undergoes increased oxidation.

	N2	AIR	UVO 5 mins	UVO 10 mins
Content of lattice oxygen	**41%**	**45%**	**67%**	**70%**
Content of oxygen vacancy	**48%**	**37%**	**25%**	**22%**
Content of absorbed oxygen	**11%**	**18%**	**8%**	**8%**

Table S1. The contents of oxygen species in MoO_x films under different treatments. The amount of lattice oxygen progressively increases from the N2 sample to the UVO-10 mins sample. The change in lattice oxygen between the UVO-5 mins and UVO-10 mins samples is minimal, indicating that the oxidized states in the MoO_x films are nearing saturation

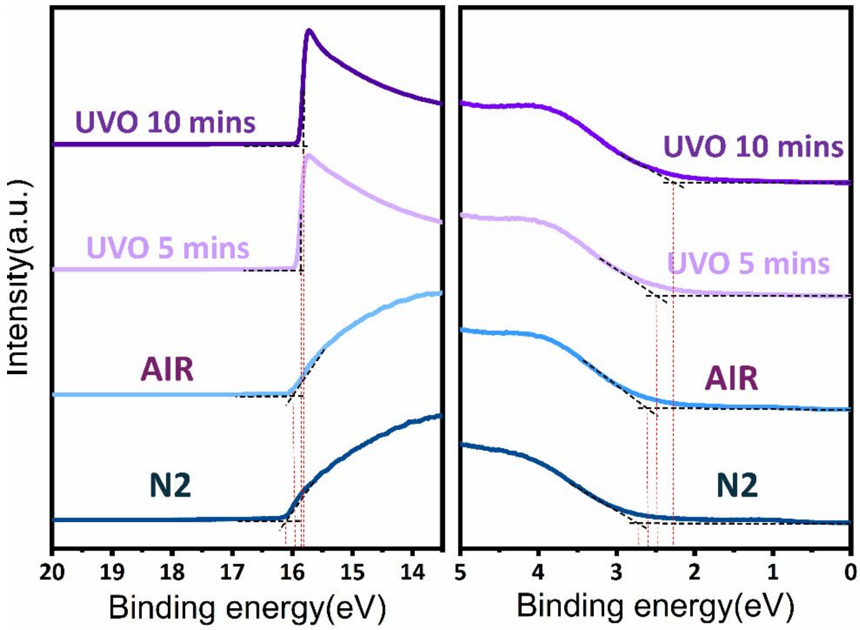

Figure S5. (Left) Photoemission cutoff of MoOx films, (Right) Valence band (VB) structure of MoOx films. The energy band will change with the oxidation states of MoO_x films.

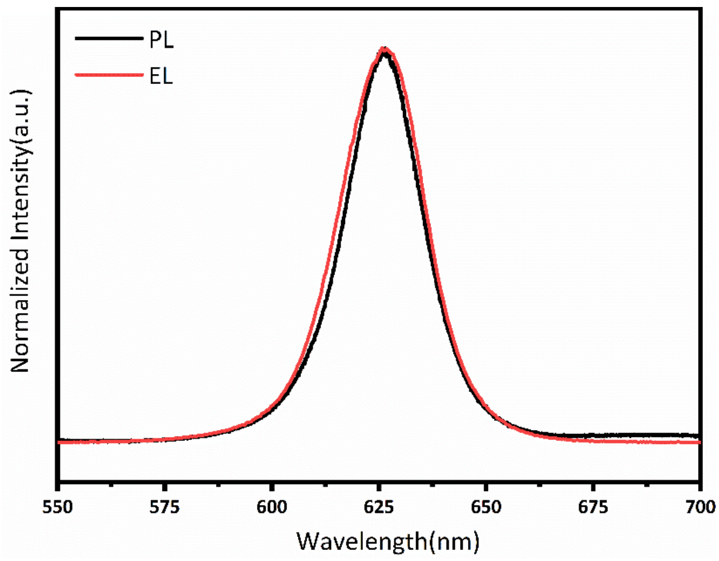

Figure S6. Normalized photoluminescence of QDs and normalized electroluminescence of the N2 MoO_x-based QLED.

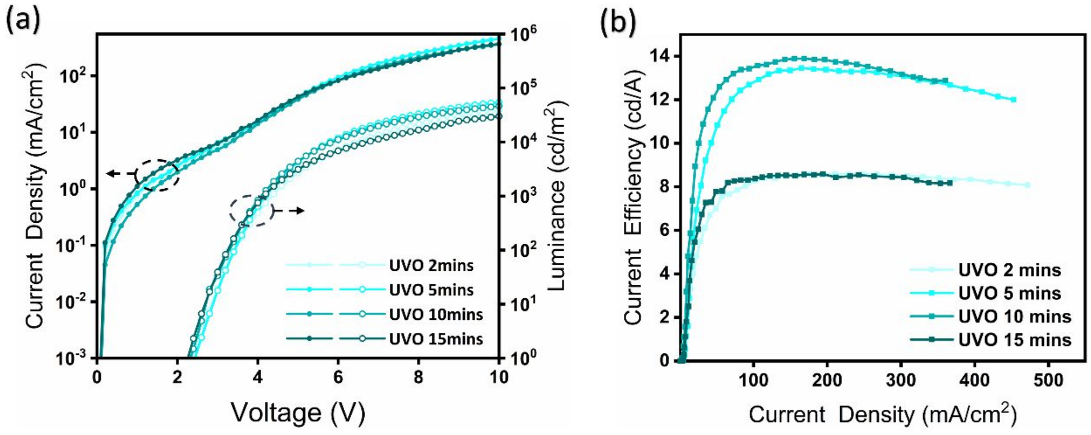

Figure S7. Device characteristics of all solution-processed QLEDs with different UV-Ozone treatment times to MoO_x layer, (a) *J-V-L*, (b) CE-*J*.

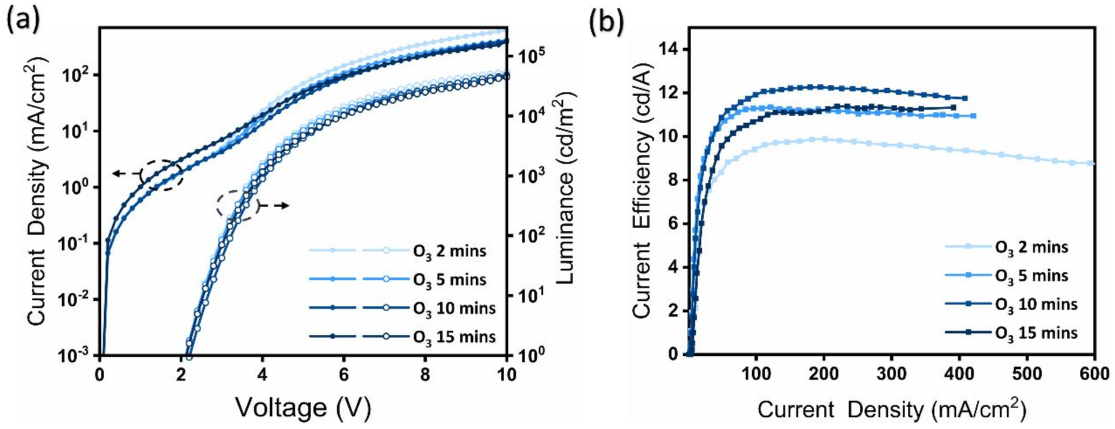

Figure S8. Device characteristics of all solution-processed QLEDs with different Ozone treatment time to MoO_x layer, (a) *J-V-L*, (b) CE-*J*.

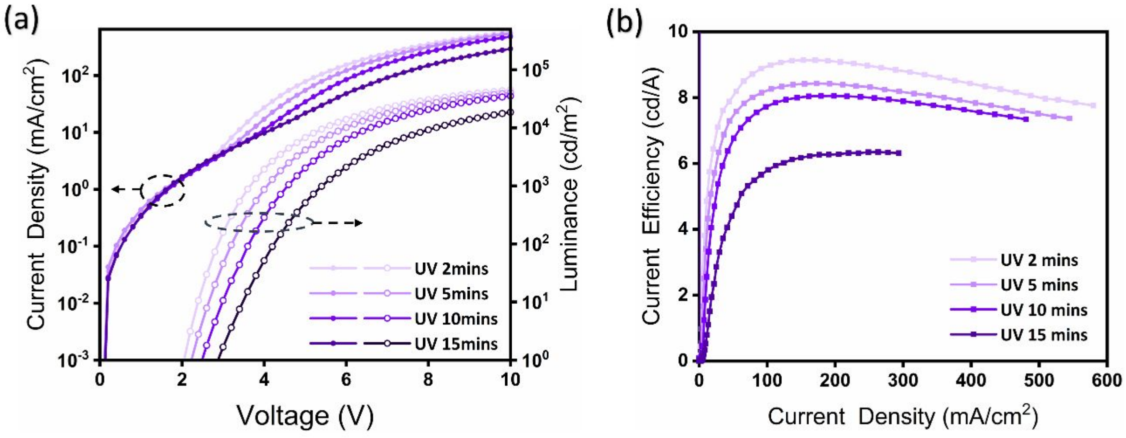

Figure S9. Device characteristics of all solution-processed QLEDs with different UV light treatment times to MoO_x layer, (a) *J-V-L*, (b) CE-*J*.

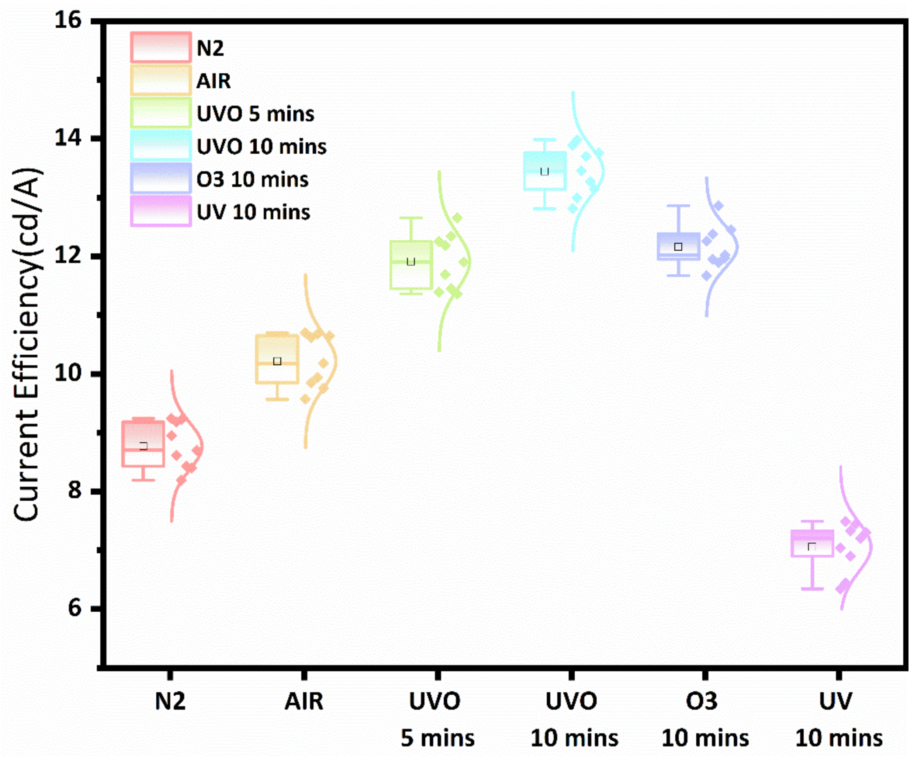

Figure S10. Box plot of CE characteristics of MoO_x-based QLEDs with different treatments to MoO_x HIL.
